# Table-Based Adaptive Digital Phase-Locked Loop for GNSS Receivers Operating in Moon Exploration Missions

**DOI:** 10.3390/s222410001

**Published:** 2022-12-19

**Authors:** Young-Jin Song, Jong-Hoon Won

**Affiliations:** Department of Electrical and Computer Engineering, Inha University, Incheon 22212, Republic of Korea

**Keywords:** GNSS receiver, adaptive digital phase-locked loop, look-up table, Moon exploration mission

## Abstract

An adaptive digital phase-locked loop (DPLL) continually adjusts the noise bandwidth of the loop filter in global navigation satellite system (GNSS) receivers to track signals by measuring the signal-to-noise ratio and/or dynamic stress. Such DPLLs have a relatively large amount of computational complexity compared with the conventional DPLL. A table-based adaptive DPLL is proposed that adjusts the noise bandwidth value by extracting it from the pre-generated table without additional calculations. The values of the noise bandwidth table are computed in an optimal manner in consideration of the thermal noise, oscillator phase noise, and dynamic stress error. The calculation method of the proper integration time to maintain the stability of the loop filter is presented. Additionally, the simulation is configured using the trajectory analysis results from the Moon exploration mission and shows that the proposed algorithm operates stably in harsh environments, while a conventional fixed bandwidth loop cannot. The proposed algorithm has a similar phase jitter performance to the existing adaptive DPLL algorithms and has an execution time that is approximately 2.4–5.4 times faster. It is verified that the proposed algorithm is computationally efficient while maintaining jitter performance.

## 1. Introduction

The digital phase-locked loop (DPLL) is widely used for the carrier tracking loop of the global navigation satellite system (GNSS) receiver to synchronize the carrier phase and frequency of the locally generated replica signal to the signal received from the antenna. The DPLL measures the carrier phase difference between the two signals and controls the numerically controlled oscillator (NCO) accordingly to track the carrier phase [1]. The characteristics of the DPLL are determined by parameters such as the loop filter order, noise bandwidth, and integration time. In general, these parameters are fixed to the specific values that are suitable for the expected operational environment of the receiver in the design phase [2]. Therefore, if the operational environment characteristics, such as the signal power and dynamic stress, become different from the expected environment, the receiver’s tracking performance is degraded, and the tracking loop may lose lock.

Furthermore, there is a trade-off in designing the parameters of the DPLL. For example, the noise bandwidth of the DPLL should be wide enough to accommodate the dynamic stress and simultaneously be sufficiently narrow to suppress the noise effect [1]. Generally, the noise bandwidth is determined considering the expected maximum line-of-sight (LOS) dynamic stress (i.e., wide enough) of the receiver. Therefore, the loop filter cannot adequately reduce the noise effect, and the phase jitter may have degraded performance [2].

In contrast, an adaptive DPLL continually measures the receiver’s current operational environment, such as the signal-to-noise ratio (SNR) and dynamic stress, and appropriately adjusts the gain of the loop filter (i.e., noise bandwidth) [3]. Consequently, the adaptive DPLL optimally tracks the incoming signal by widening the noise bandwidth during high dynamic stress while reducing the phase jitter by narrowing the noise bandwidth when the signal power is weak or interference has occurred.

Performance analysis and optimization of the generalized DPLL and digital frequency-locked loop (DFLL) based on the minimum mean square error criterion are presented in [4]. They proposed an adaptive DPLL and DFLL algorithm that automatically adjusts the integration time and noise bandwidth of the loop filter for optimal performance. The simulation, which uses scenarios of high and low dynamic stress with a time-varying carrier-to-noise-density ratio (C/N_0_), showed that the proposed adaptive DPLL is superior to the traditional fixed-gain DPLL. However, the proposed algorithm uses only the C/N_0_ value as an input, so the dynamic performance of the adaptive loop filter is fixed. An adaptive DPLL algorithm for high dynamic applications is suggested in [5] and is verified to have a better performance compared to the traditional DPLL using simulation.

The fast adaptive bandwidth (FAB) algorithm is proposed in [6], which measures the C/N_0_ and dynamic stress to calculate the optimal noise bandwidth for the current environment by estimating the pole location of the loop transfer function that minimizes the thermal noise. Adaptive DPLL algorithms, including FAB, fuzzy logic (FL), and loop-bandwidth control algorithm (LBCA), are evaluated and compared in terms of the performance and computational complexity for the various scenarios in [3].

An adaptive DPLL, where the loop gain is controlled adaptively, is presented in [7]. Mathematical models of second- and third-order DPLL with each error characteristic are derived and analyzed for various dynamics and signal strength. Experiments are performed for the proposed method with a conventional DPLL, DFLL-assisted DPLL, and FAB-based DPLL. Results show that the proposed one operates well in a highly dynamic scenario. LBCA-based adaptive carrier tracking loop for robust tracking in a sounding rocket is proposed in [8]. The LBCA is extended to the DFLL-assisted DPLL structure to improve the robustness of the tracking loop. Simulation results verified the reliable performance of the proposed algorithm during the sounding rocket launch scenario.

In addition, adaptive DPLLs using the Kalman filter (KF) are studied in [9,10,11,12]. The key idea of [9] is to properly adjust the equivalent noise bandwidth of the signal tracking KF with the empirical knowledge of the C/N_0_ and the signal dynamics. The tuning method for the signal tracking KF using the relationship between the C/N_0_ and the equivalent noise bandwidth is introduced in [10]. An adaptive KF structure that adjusts the measurement and process noise covariance (which are fixed in the case of the standard KF) is presented in [11].

These adaptive DPLL algorithms may operate well while maintaining tracking performance even in harsh environments such as low signal power and high dynamic stress. Using the adaptive DPLL for the ionospheric scintillated environment is investigated in [11,12]. Simulation results showed that the adaptive algorithms outperforming the traditional DPLL during the ionospheric scintillation occurrence.

Most of the aforementioned adaptive DPLLs that adopt an optimal manner have a relatively larger computational burden when compared with the traditional DPLL. A projected bandwidth method that uses a look-up table for the determination of noise bandwidth is proposed in [2]. This algorithm does not utilize dynamic stress estimation to reduce the computational burden; therefore, it inevitably has suboptimal performance. Similarly, look up table-based low-complexity adaptive carrier tracking loop is presented in [13]. It uses the direct-state KF for the DPLL combined with the DFLL as the carrier tracking loop filter for robustness. The look-up table approach is utilized to reduce computational complexity. Consequently, the proposed structure has superior dynamic tracking performance and low complexity simultaneously.

Moreover, although the GNSS was originally designed for users and applications in terrestrial regions, such as land, maritime, and air, the GNSS signal can also be used in deep space. The space service volume (SSV) is defined as a shell extending from 3,000 km to 36,000 km in altitude [14]. For the region with an altitude of less than 8,000 km, the signal reception environment is similar to that of the ground users, which indicates that the GNSS receiver does not operate with much difficulty. In contrast, receivers that operate in the higher regions are either near or beyond the satellites. For this reason, these receivers should obtain satellite signals from the nadir direction, which has weak signal power due to the large free-space loss [15].

In addition, the number of visible satellites of the GNSS receivers at such high altitudes is decreased because the Earth blocks most of the main-lobe signal of the transmitter antenna pattern. Consequently, the receivers can only receive the spilled-over signal of the main lobe, which is the reason for the reduced visible satellite numbers. To counter this problem, studies that effectively use the side-lobe signal of a transmitter antenna have been performed [15,16,17,18]. The results showed that the number of visible satellites is increased by utilizing the side-lobe signal. However, the side-lobe signal inherently has a relatively small transmitting gain compared with the main lobe signal, which results in further decreases in the received signal power at the SSV receivers.

Additionally, several studies have been performed to assess the feasibility of GNSS signals for Moon exploration [19,20,21,22,23]. In particular, GNSS-based navigation during lunar exploration has advantages such as fewer ground operations, reduced costs, and decreased payload weight and volume [20]. However, GNSS receivers suffer weakened signal power and high dynamic stress during the transfer orbit mission. The dedicated receiver design and verification for such a challenging environment are presented in [21,22,23,24].

This paper complements and expands the interim result presented in [25]. A computationally-efficient table-based adaptive DPLL algorithm for GNSS receivers operating in Moon exploration missions is proposed. Each noise bandwidth value of the table is calculated optimally in consideration of thermal noise, Allan deviation, vibration-induced oscillator phase noise, and dynamic stress error in the receiver design phase. The simulation scenario is configured using the analysis result of the mission trajectory for the Moon exploration, and the numerical simulation result for the proposed algorithm is presented. This algorithm is compared with other adaptive DPLL algorithms (FAB, FL, LBCA) in terms of jitter performance and execution time.

The remainder of this paper is composed as follows: The Moon exploration mission trajectory is analyzed in Section 2. The calculation method and generation result example of the optimal bandwidth table are presented in Section 3. The proposed table-based adaptive DPLL algorithm is illustrated in Section 4, and the numerical simulation result is discussed in Section 5. The proposed algorithm is evaluated in Section 6, followed by the conclusion in Section 7.

## 2. Moon Exploration Mission Trajectory

**Figure 1:**
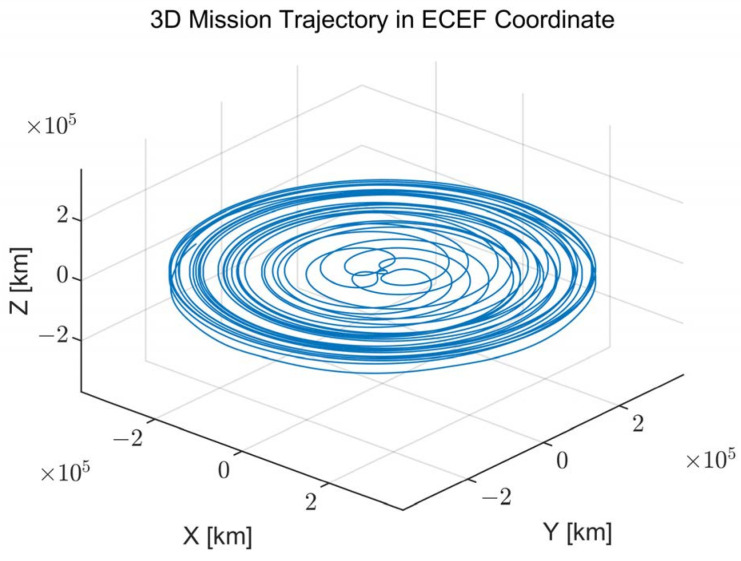
Overall Moon exploration mission trajectory in the earth-centered, earth-fixed (ECEF) coordinate.

**Figure 2:**
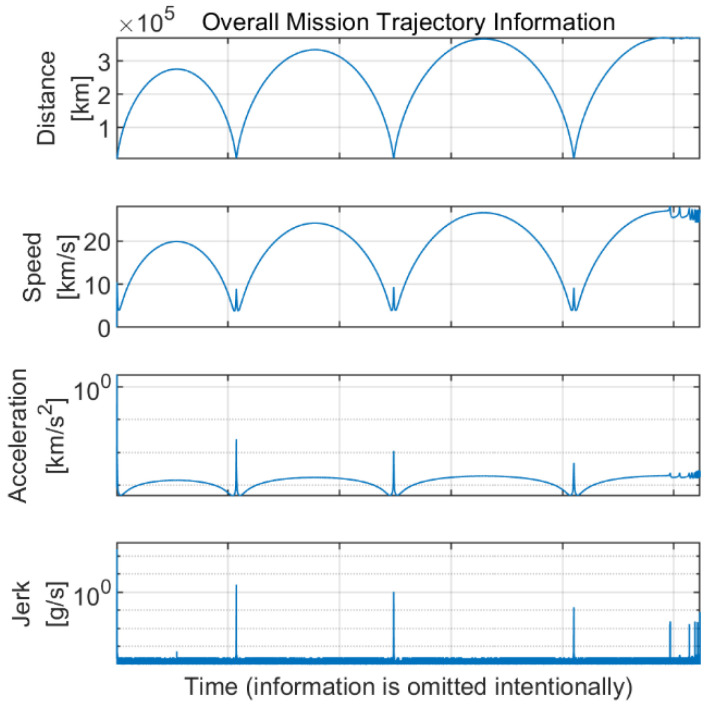
Overall information on the Moon exploration spacecraft during the mission trajectory.

The mission trajectory of the spacecraft for the exploration of the Moon is presented and analyzed in this section. The overall mission trajectory in the earth-centered, earth-fixed (ECEF) coordinate is presented in Figure 1. The Earth is located at the origin point of the coordinate, and the Moon exploration spacecraft maneuvers toward the Moon. The spacecraft is assumed to be pointing to Earth during the maneuver (i.e., no attitude maneuver), indicating that the GNSS antenna is always facing the Earth. The mission trajectory has a gradually increasing circle-like shape. The distance, speed, acceleration, and jerk values of the spacecraft during the Moon exploration mission are presented in Figure 2. From this figure, the distance of the spacecraft is depicted as several arc shapes with respect to time, while the height of the arc gradually increases. Additionally, large acceleration and jerk occur when the distance becomes close to Earth due to the strong propulsion power of the spacecraft, which is used for advancing further away.

**Figure 3:**
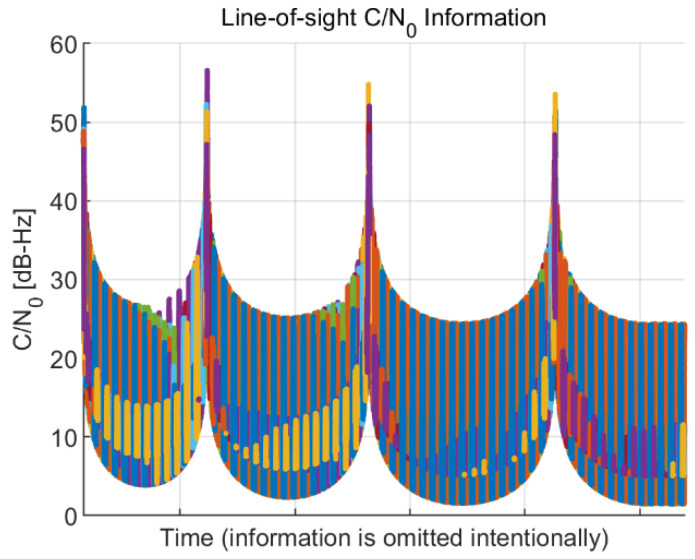
Line-of-sight (LOS) carrier-to-noise-density ratio (C/N_0_) information of the Moon exploration spacecraft during the mission trajectory. Each color in the figure indicates a different global positioning system (GPS) satellite.

**Figure 4:**
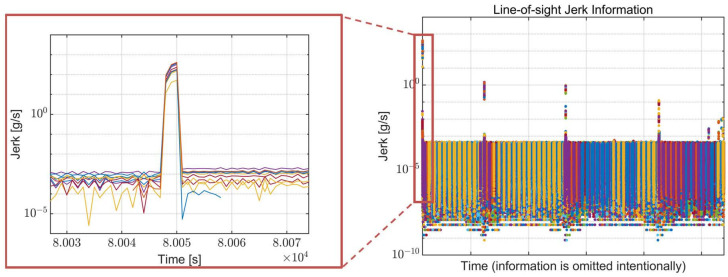
LOS jerk information of the Moon exploration spacecraft during the mission trajectory. Each color in the figure indicates a different GPS satellite.

LOS information, such as the C/N_0_ and jerk value for each visible satellite, is calculated for the global positioning system (GPS) using the GPS orbit information and presented in Figure 3 and Figure 4, respectively. For the calculation of the LOS information, the side-lobe signal of the transmitter antenna pattern is utilized. Each color in the figures indicates a different GPS satellite. As expected, the C/N_0_ information in Figure 3 has a reversed arc shape because the free space loss is increased when the distance between the spacecraft and the GPS satellites increases. The C/N_0_ has a range from 1.48 to 56.58 dB-Hz. The calculated overall LOS jerk information and the enlarged version of the maximum jerk region are presented in Figure 4. The jerks occur periodically due to the propulsion power of the spacecraft, and the magnitude of each jerk gradually decreases. The maximum jerk occurs at the beginning of the trajectory, and its magnitude is approximately 411 g/s, remaining for only 1–2 s.

Figure 3 and Figure 4 show that the low SNR circumstance and high dynamics condition do not coexist during the mission trajectory. In other words, when the receiver suffers low signal power, there is negligible dynamic stress to the receiver, and when high dynamic stress exists, the signal power is sufficiently high. These analysis results are taken into account later when configuring the simulation scenario.

## 3. Optimal Bandwidth Table

The proposed table-based adaptive DPLL algorithm (which will be discussed in Section 4) requires a pre-computed optimal bandwidth table that contains an optimal noise bandwidth value for each C/N_0_ and dynamic stress. The optimal noise bandwidth is calculated using the theoretical measurement error modeling of the DPLL that incorporates the thermal noise, Allan deviation, vibration-induced oscillator phase noise, and dynamic stress error. This paper assumes that the order of the DPLL is third-order to effectively track the dynamics components leading up to acceleration. In addition, the receiver is assumed to track the pure pilot channel (i.e., phase transition due to the navigation data bit or secondary code does not exist) to track very weak signals such that the C/N_0_ value is as low as a few dB-Hz. However, the other orders, including the data channel case, can be applied by an analogous method.

The overall 1-sigma measurement error of the pilot DPLL is modeled as follows [1]:

(1) $$\sigma_{DPLL}=\sqrt{\sigma_{t}^{2}+\theta_{A}^{2}+\sigma_{v}^{2}}+\frac{\theta_{e}}{3}\leq 30\;\deg$$

where $\sigma_{t}$ is the thermal noise [deg], $\theta_{A}$ and $\sigma_{v}$ are the Allan deviation and vibration-induced oscillator phase noise [deg], respectively, and $\theta_{e}$ is the LOS dynamic stress error [deg]. The rule-of-thumb for the tracking threshold is that the overall 3-sigma error must not exceed 1/4 of the pull-in range [1]. Because the pull-in range of the pilot DPLL discriminator is 360 degrees, the threshold of the 3-sigma error is 90 degrees, which indicates that the 1-sigma error must not exceed 30 degrees.

The thermal noise of the pilot DPLL is modeled as follows [1]:

(2) $$\sigma_{t}=\frac{360}{2\pi }\sqrt{\frac{B_{n}}{C/N_{0}}}\;\left[\deg\right]$$

where $B_{n}$ is the noise bandwidth [Hz] of the loop filter and $C/N_{0}$ is the carrier-to-noise density ratio converted to a linear scale [Hz]. Notably, the squaring loss cannot be found in (2) because this equation is modeled for the pilot channel. Because (2) shows that the thermal noise is proportional to the noise bandwidth, the noise bandwidth should be narrowed to reduce the thermal noise.

The Allan deviation oscillator phase noise of the third-order DPLL is modeled as follows [26]:

(3) $$\theta_{A}=\frac{360}{2\pi }\sqrt{2\pi^{2}f_{c}^{2}\left(\frac{\pi^{2}h_{-2}}{3\omega_{0}^{3}}+\frac{\pi h_{-1}}{3\sqrt{3}\omega_{0}^{2}}+\frac{h_{0}}{6\omega_{0}}\right)}\;\left[\deg\right]$$

where $f_{c}$ is the carrier frequency [Hz] of the oscillator; $\omega_{0}$ is the natural frequency [Hz] of the loop filter, which can be calculated using a typical value for the third-order loop ($\omega_{0}=1.27B_{n}$); and $h_{0}$ [s], $h_{-1}$ [s/s], and $h_{-2}$ [s/s^2^] are the clock parameters related to the Allan deviation. In this work, the oven-controlled crystal oscillator (OCXO) is assumed to be used for the spacecraft. The corresponding clock parameters used for the paper are presented in Table 1.

The vibration-induced oscillator phase noise of the band-limited vibration with constant power spectral density (PSD) for the third-order DPLL is modeled as follows [26]:

(4) $$\sigma_{v}=\frac{360}{2\pi }\sqrt{\frac{2\pi f_{c}^{2}k_{g}^{2}G_{g}}{\omega_{0}}\left[\begin{array}{c} \frac{1}{3}\left(\arctan\frac{\omega_{2}}{\omega_{0}}\;-\;\arctan\frac{\omega_{1}}{\omega_{0}}\right)\;+\;\frac{1}{4\sqrt{3}}\ln\frac{\left(\omega_{0}^{2}\;-\;\omega_{0}\omega_{2}\sqrt{3}\;+\;\omega_{2}^{2}\right)\left(\omega_{0}^{2}\;+\;\omega_{0}\omega_{1}\sqrt{3}\;+\;\omega_{1}^{2}\right)}{\left(\omega_{0}^{2}\;+\;\omega_{0}\omega_{2}\sqrt{3}\;+\;\omega_{2}^{2}\right)\left(\omega_{0}^{2}\;-\;\omega_{0}\omega_{1}\sqrt{3}\;+\;\omega_{1}^{2}\right)}\\ +\frac{1}{6}\left(\arctan\frac{-\sqrt{3}\omega_{0}+2\omega_{2}}{\omega_{0}}+\arctan\frac{\sqrt{3}\omega_{0}+2\omega_{2}}{\omega_{0}}-\arctan\frac{-\sqrt{3}\omega_{0}+2\omega_{1}}{\omega_{0}}-\arctan\frac{\sqrt{3}\omega_{0}+2\omega_{1}}{\omega_{0}}\right) \end{array}\right]}[\deg]$$

where $k_{g}$ is the oscillator’s g-sensitivity [parts/g], $G_{g}$ is the single-sided vibration spectral density [g^2^/Hz], and $\omega_{1}$ and $\omega_{2}$ are the lower and upper limits [rad/s] of the constant vibration PSD, respectively. Additionally, the parameter values for the vibration-induced oscillator phase noise calculation are selected based on the assumption that the receiver uses a high-quality oscillator and suffers a moderate amount of vibration. These parameter values are presented in Table 2.

The LOS dynamic stress error of the third-order DPLL is modeled as follows [1]:

(5) $$\theta_{e}=\frac{R^{\prime \prime \prime }}{\omega_{0}^{3}}\;\left[\deg\right]$$

where $R^{\prime \prime \prime }$ is the LOS jerk dynamic stress [deg/s^3^]. Since the third-order DPLL can track up to the acceleration, the dynamic stress error of the third-order DPLL is induced by only the jerk value. Notably, the dynamic stress error is inversely proportional to the natural frequency of the loop filter, so the noise bandwidth (which is proportional to the natural frequency) should be widened to reduce the dynamic stress error.

The overall root mean square error (RMSE) of the DPLL measurement error presented in (1) has a trade-off with respect to the noise bandwidth. In other words, a simple selection of the noise bandwidth to a low or high value can increase the measurement error, and as a result, the performance of the DPLL may be degraded. Therefore, the optimal bandwidth is selected as a point that minimizes the RMSE as follows:

(6) $$B_{opt}=\underset{B_{n}\in \left(0,\infty \right)}{\arg\;\min}\sigma_{DPLL}$$

Although the optimal bandwidth can be found, the resulting RMSE can exceed the rule-of-thumb tracking threshold (i.e., 30 degrees for the pilot DPLL as shown in (1)) because the operating condition can be too harsh (e.g., very low SNR, very high dynamics, low oscillator quality, etc.). In that case, the calculated bandwidth is not saved to the optimal bandwidth table because the stable operation of the DPLL cannot be ensured theoretically, and the usage of the calculated value may yield the divergence of the loop filter. The minimum RMSE determined by the calculated optimal bandwidth is expressed as follows:

(7) $$\sigma_{min}=\underset{B_{n}\in \left(0,\infty \right)}{\min}\sigma_{DPLL}={\left.\sigma_{DPLL}\right|}_{B_{n}=B_{opt}}$$

**Figure 5:**
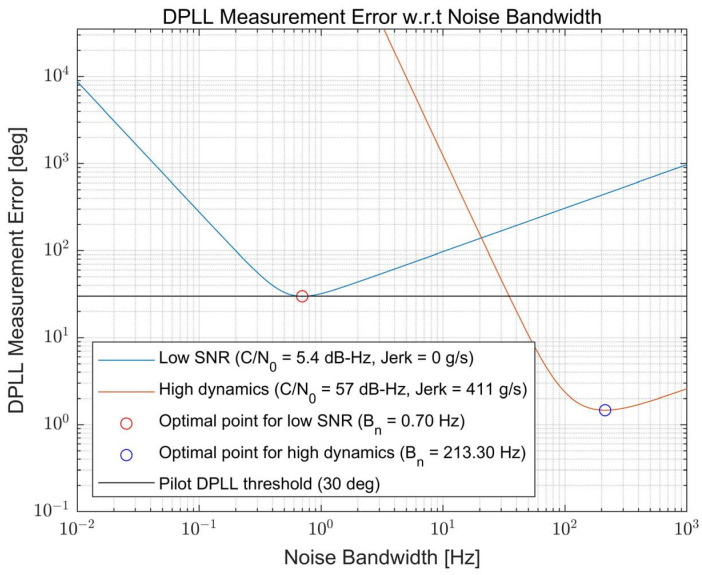
Example of the measurement error calculation results with respect to the noise bandwidth variation for low signal-to-noise ratio (SNR) and high dynamics conditions.

Examples of the overall measurement error calculation results for the extreme cases, namely low SNR and high dynamics conditions, are illustrated in Figure 5. Each condition is selected in consideration of the analysis result obtained from Section 2 where the maximum value of the C/N_0_ and the jerk are selected to be similar to the analysis result. However, the minimum value of the C/N_0_ is selected as 5.4 dB-Hz (not 1.48 dB-Hz, as can be observed in Figure 3) because it is the smallest C/N_0_ that the receiver can track in a theoretical sense (i.e., the smallest C/N_0_ where the theoretical measurement error is less than 30 degrees). The figure shows that the measurement error varies with the noise bandwidth value, and one optimal point at which the measurement error is minimized exists for each condition. As expected, the optimal bandwidth for the low SNR condition is very narrow ($B_{n}$ = 0.7 Hz) to suppress the large noise effect due to the very low C/N_0_ of 5.4 dB-Hz. Furthermore, the optimal bandwidth is very wide ($B_{n}$ = 213.3 Hz) for the case of the high dynamics to track fast variations in the signal component.

The algorithm that generates the optimal bandwidth table is presented in Algorithm 1. This algorithm starts with the initialization of setting the required parameters for the calculation of the DPLL measurement error. Then, the optimal bandwidth and the minimum RMSE at the optimal point are calculated for each C/N_0_ and jerk value. If the minimum RMSE is less than 30 degrees, the optimal bandwidth is saved to the optimal bandwidth table; otherwise, the calculated value is discarded. A two-dimensional table is outputted by the algorithm and is used for the table-based adaptive DPLL algorithm.
**Algorithm 1** Optimal bandwidth table generation algorithm.1:**Initialization:**2:   Set target range of $C/N_{0}$ and jerk $R^{\prime \prime \prime }$
3:   Set carrier frequency $f_{c}$
4:   Set clock parameters $h_{0}$, $h_{-1}$, $h_{-2}$
5:   Set parameters of vibration-induced oscillator phase noise $k_{g}$, $G_{g}$, $\omega_{1}$, $\omega_{2}$
6:**Optimal bandwidth calculation:**7:   **for** each $C/N_{0}$ **do**8:      **for** each $R^{\prime \prime \prime }$ **do**9:         Calculate $B_{opt}$ and $\sigma_{min}$ for current $C/N_{0}$ and $R^{\prime \prime \prime }$
10:         **if** $\sigma_{min}<30$ **then**11:            Save $B_{opt}$ to optimal bandwidth table12:         **else**
13:            Do not save *B_opt_*
14:         **end if**
15:      **end for**
16:   **end for**


**Figure 6:**
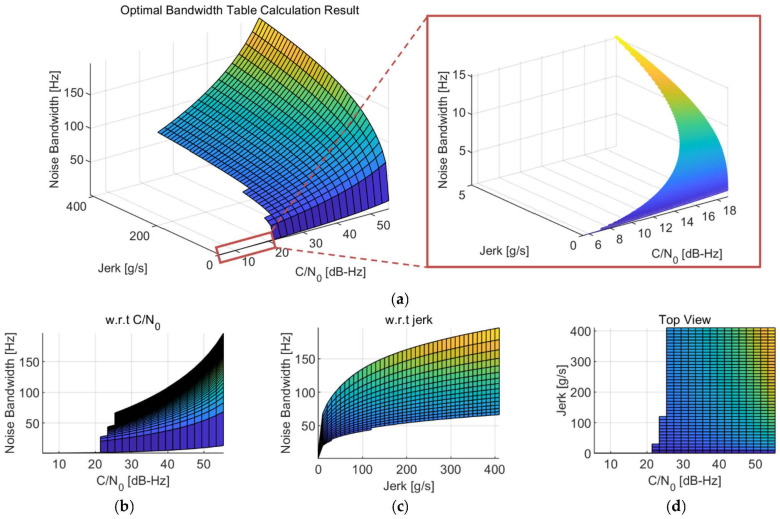
Result example of the generated optimal bandwidth table: (**a**) Overall shape; (**b**) Optimal bandwidth with respect to the C/N_0_; (**c**) Optimal bandwidth with respect to the jerk; (**d**) Top view.

The generated optimal bandwidth table for the Moon exploration mission, as an example, is presented in Figure 6. Note that another table can be generated for another operating environment of the GNSS receiver with modified parameters. For the current scenario, the optimal bandwidth table was generated with the step of 0.1 dB-Hz and 1 g/s for the C/N_0_ and the jerk, respectively. The target generation ranges for each bin were selected as the maximum expected values (i.e., 57 dB-Hz for C/N_0_ and 411 g/s for jerk) for the operating environment. Figure 6a shows the overall shape of the calculated optimal bandwidth table in a three-dimensional view, as the optimal bandwidth for each bin is represented as a height in the figure. The same shape is presented in Figure 6b,c with respect to the C/N_0_ and jerk, respectively. A top view of the optimal bandwidth table is illustrated in Figure 6d. Since the optimal bandwidth is not saved to the optimal bandwidth table if the minimum RMSE exceeds 30 degrees, empty spaces can be observed in Figure 6d. Therefore, the empty space means the receiver cannot operate in that condition in a theoretical sense.

The optimal bandwidth table utilizes the thermal noise, Allan deviation, vibration-induced oscillator-phase noise, and dynamic stress error for the bandwidth calculations as these are well-known error sources of DPLL. Parameter values for each error source, especially for the oscillator, are fixed to the expected values in the operating environment of the receiver. Since the environment is the Moon exploration mission in this study, these values are set as the OCXO values suffering a moderate amount of vibration with assumptions as presented in Table 1 and Table 2. However, in real conditions, they cannot be the same as the expected ones and would be changed as the environment changes. Thus, the optimality can only be assured when the environment is similar to the expected one, and if it deviates greatly, an additional estimator for the oscillator phase noise is required.

## 4. Table-Based Adaptive DPLL

**Figure 7:**
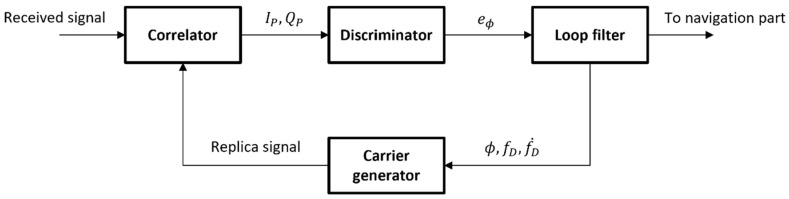
Simplified structure of conventional carrier tracking loop.

A simplified structure of a conventional carrier tracking loop of the GNSS receiver is shown in Figure 7. Note that the code tracking components are omitted in the figure for the simplicity of discussion. The carrier tracking process starts at the correlator. The correlator correlates the received signal transferred from the radio frequency front-end and the replica signal produced in the carrier generator. Then, the discriminator measures the difference ($e_{\phi }$) of the carrier phase between the two signals using the in-phase and quadrature prompt correlator outputs ($I_{P}$, $Q_{P}$), which are inputted to the discriminator. The discriminator output is filtered in the loop filter, with the loop filter also estimating the differential components that depend on the loop filter order: the carrier phase (ϕ), Doppler frequency ($f_{D}$), and Doppler rate ($\dot{f_{D}}$) in the case of the third-order DPLL. Finally, the carrier generator and the NCO (which is omitted in the figure) generate a new replica signal to close the loop.

**Figure 8:**
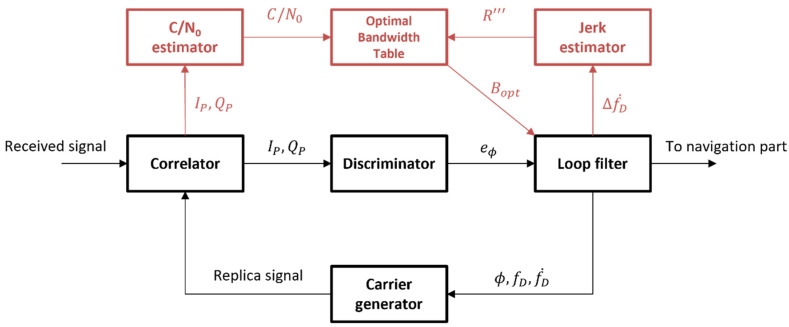
Structure of the proposed table-based adaptive digital phase-locked loop (DPLL) algorithm.

The structure of the proposed table-based adaptive DPLL is presented in Figure 8. The optimal bandwidth table, the C/N_0_ estimator, and the jerk estimator are newly added to the conventional structure. The C/N_0_ estimator estimates the C/N_0_ of a current channel using a C/N_0_ estimation algorithm (such as the variance-summing method or power-ratio method explained in [27]). As presented in [5], the LOS jerk is estimated in the jerk estimator by differencing the adjacent Doppler rate, which is estimated in the loop filter with a time interval. The optimal bandwidth table returns the optimal bandwidth of the current environment efficiently using the pre-generated table with a slight calculation for finding the index of the table.

Since the immediate and rapid change in the noise bandwidth can incur the instability of the tracking loop, the optimal bandwidth is reflected smoothly as follows:

(8) $$B_{n}\left[k+1\right]=\alpha B_{opt}+\left(1-\alpha \right)B_{n}\left[k\right]$$

where α is a coefficient for a smooth transition and is heuristically set to 0.1.

The DPLL is stable if the normalized bandwidth, which is a product between the noise bandwidth and the integration time, is less than one (especially when the normalized bandwidth is close to zero) because the corresponding analog counterpart can be correctly represented [28]. The proper value of the integration time is calculated in consideration of this condition as follows:

(9) $$T=\Delta T\times \lfloor \frac{BT_{norm}}{\Delta T\times B_{n}}\rfloor \;\left[s\right]$$

where ⌊·⌋ is a floor function, ΔT is a minimum unit value [s] for the variation in the integration time and $BT_{norm}$ is the pre-defined target normalized bandwidth [unitless]. Here, ΔT is used to prevent excessively frequent changes in the integration time value, which can be a reason for the loop filter’s instability. Considering this, ΔT is set to 20 ms, so the integration time is changed for the step of 20 ms. $BT_{norm}$ is set to 0.3, which is identical to the typical value that can be obtained from the noise bandwidth of 15 Hz and the integration time of 20 ms for the GPS L1 C/A receiver. Consequently, the normalized bandwidth of the table-based adaptive DPLL is maintained at values below the $BT_{norm}$, if possible. Sometimes, the integration time value of zero can be obtained from (9) if the noise bandwidth is relatively large. In that case, the lower bound condition is applied, which sets the integration time as the length of one spreading code period as follows:

(10) $$T=\left\{\begin{array}{c} \;T,\;\;\;T>0\\ T_{code},\;\;T=0 \end{array}\right.$$

where $T_{code}$ is spreading code period [s] (e.g., 1 ms for GPS L1 C/A).

The proposed table-based adaptive DPLL algorithm is presented in Algorithm 2. The algorithm corresponds to the one iteration procedure of the carrier tracking loop, repeating for every tracking loop operation interval. The algorithm starts with the reception of the estimated C/N_0_ and jerk values from each estimator. The matching index of the optimal bandwidth table is determined, and the corresponding optimal bandwidth value is extracted from the table. Since the optimal bandwidth value is saved only if the minimum RMSE does not exceed the threshold, the existence of the optimal bandwidth value means the receiver can theoretically operate in the current condition. Therefore, the optimal bandwidth is applied to the noise bandwidth through (8); otherwise, the recent value of the noise bandwidth is maintained. Finally, the new noise bandwidth is handed over to the loop filter, and the integration time for the next integration is calculated using (9).
**Algorithm 2** Table-based adaptive DPLL algorithm.1:Get estimated $C/N_{0}$ and $R^{\prime \prime \prime }$
2:**Noise bandwidth transition:**3:   Find index for $C/N_{0}$ and R′′′
4:   Get $B_{opt}$ from optimal bandwidth table5:   **if** $B_{opt}$ is exist **then**6:      $B_{n}\left[k+1\right]=\alpha B_{opt}+\left(1-\alpha \right)B_{n}\left[k\right]$
7:   **else**
8:      $B_{n}\left[k+1\right]=B_{n}\left[k\right]$
9:   **end if**
10:   Return $B_{n}\left[k+1\right]$ to the loop filter11:**Integration time calculation:**12:   $T=\Delta T\times BT_{norm}/\left(\Delta T\times B_{n}\right)$
13:    Set next integration time to T


As one can anticipate, the performance of this algorithm is dependent on the performance of the estimators. If the estimated C/N_0_ and jerk are wrong, the resulting noise bandwidth is not optimal anymore. Therefore, the algorithm fits the environment that has slow variations of C/N_0_ and/or jerks where the estimators can estimate them without much difficulty. Additionally, scenarios in which the trajectory is fixed so that the receiver can know its approximate location and signal reception characteristics are also appropriate conditions for the algorithm. The Moon exploration mission corresponds to both conditions. Dedicated estimators would be required to apply the algorithm to other conditions, such as having fast variations, etc.

## 5. Simulation

**Figure 9:**
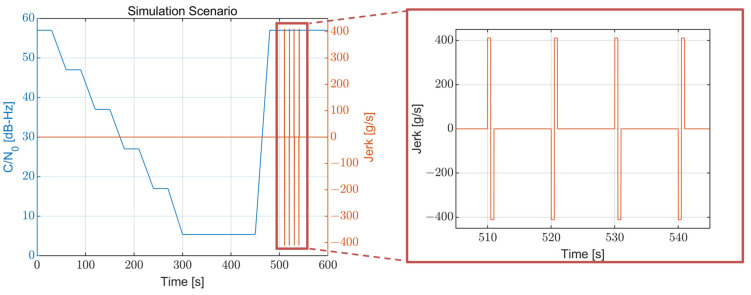
Configured simulation scenario information. The C/N_0_ has a range of 5.4–57 dB-Hz and the maximum jerk is 411 g/s.

The simulation scenario is configured using the analysis results for the Moon exploration mission trajectory obtained in Section 2. The configured simulation scenario is shown in Figure 9. The overall length of the simulation is 600 s. The C/N_0_ starts from 57 dB-Hz and decreases to 5.4 dB-Hz for 300 s. The value of 5.4 dB-Hz is selected as a minimum C/N_0_ because it is the minimum C/N_0_ that allows the receiver to operate, which is obtained from the optimal bandwidth table generation procedure in Section 3. The C/N_0_ decreases with a repeated uniform pattern that maintains its value for 30 s and decreases by 10 dB-Hz for 30 s. Furthermore, the C/N_0_ maintains 5.4 dB-Hz starting from 300 s for 150 s and then quickly recovers to 57 dB-Hz at 450 s. After the SNR has recovered, the high dynamics region begins; the jerk dynamic stresses occur four times, starting from 510 s with an interval of 10 s. Each of these occurrences is set to a magnitude of 411 g/s and a length of 1 s because the observed jerk dynamic stress in the Moon exploration mission lasted only for 1–2 s.

The simulation was configured like this because the Moon exploration mission has a critical characteristic, as can be observed from Section 2. The very strong jerk dynamic stress only occurs at the high C/N_0_ region, so the low signal power and high dynamic stress do not coexist in our situation. Therefore, the low SNR region and high jerk region were intentionally separated in the simulation to imitate such mission conditions. From this point, it is thought that the configured simulation scenario well reflects the characteristics of the mission, and the harshness of both situations is similar.

The other signal components for generating the simulation signal, such as the code delay, carrier phase, Doppler frequency, and Doppler rate, are generated by integrating the configured jerk value. The starting values for integrating the Doppler frequency and the Doppler rate are set to the mean values obtained from the mission trajectory information of the Moon exploration (mean Doppler frequency: 7744.03 Hz, mean Doppler rate: 2.33 Hz/s). The simulation signal is generated according to the generated signal components and processed by the software receiver. The properties for the GNSS signal generation are set identically to that of the GPS L5 pilot signal with an assumption of the synchronized secondary code (i.e., no phase transition). Since the GPS L5 signal has a larger off-nadir angle (26 degrees) than the GPS L1 signal (23.5 degrees), the SSV users benefit due to the larger main-lobe beamwidth [29,30].

The tracking loop of the receiver is composed of the code tracking loop and carrier tracking loop. The code tracking loop is set to the carrier-aided first-order delay-locked loop (DLL) with the noncoherent early-minus-late discriminator. The noise bandwidth of the DLL is fixed at 1 Hz, which is a very narrow bandwidth. However, because it is aided by the carrier tracking loop, most of the dynamics can still be captured. The carrier tracking loop is set to the third-order DPLL with the four-quadrant arctangent discriminator and uses the proposed table-based adaptive algorithm. The DFLL is intentionally disconnected in the carrier tracking loop to focus on the DPLL.

**Figure 10:**
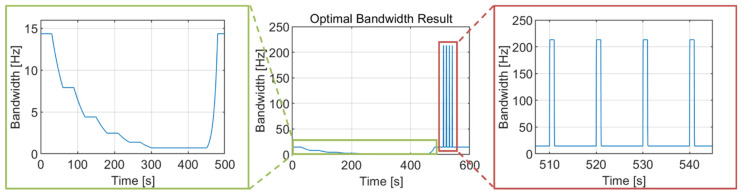
Optimal bandwidth variation during the simulation. The noise bandwidth is narrowed to 0.7 Hz as the C/N_0_ is lowered to 5.4 dB-Hz and widened to 213.3 Hz as the jerk dynamic stress increases to 411 g/s.

The noise bandwidth variation, which uses the optimal bandwidth table during the simulation, is presented in Figure 10. As intended, the selected optimal bandwidth is decreased for the first 300 s as C/N_0_ decreases. From 300 s to 450 s, the noise bandwidth is set to 0.7 Hz, which is very narrow to suppress the noise effect. When the jerk dynamic stress exists after 500 s, the noise bandwidth is widened to 213.3 Hz to track the high dynamics of signal components.

**Figure 11:**
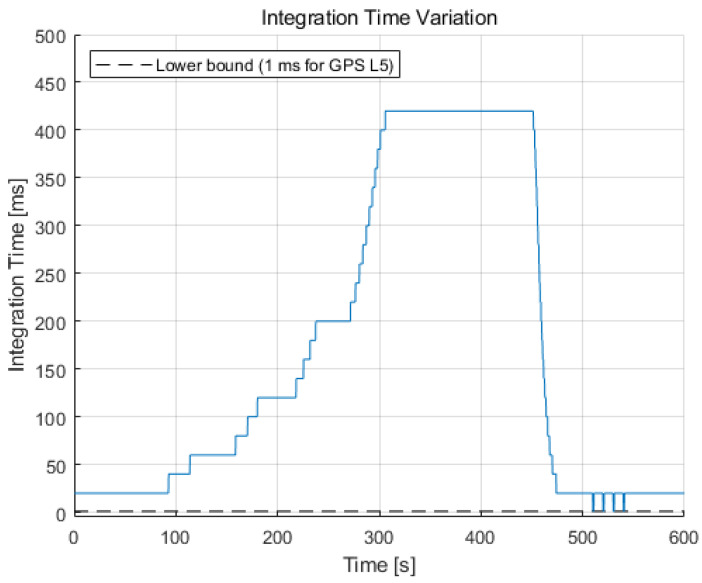
Integration time variation during the simulation varied with a step of ΔT (20 ms for this study). It increases to 420 ms for the C/N_0_ of 5.4 dB-Hz and reduces to the lower bound value in the high dynamics region.

The integration time variation during the simulation is illustrated in Figure 11. This variation has an almost inversely proportional shape with the noise bandwidth of Figure 10 to maintain a constant normalized bandwidth value, varying with a step of ΔT (20 ms for this study). The integration time increases to 420 ms for 300–450 s because the noise bandwidth is narrowed to 0.7 Hz in that region. For the high dynamics region (after 500 s), the integration time reduces to 1 ms, which is a lower bound for the GPS L5 signal when the jerk dynamic stress exists.

In fact, tracking loops with a long integration time, such as a few hundred milliseconds, are vulnerable to the instability of the oscillator, because the low-quality oscillator fluctuates during the long integration period. Therefore, to extend the integration time, a feasibility analysis for the target integration time and the oscillator quality should be done. It is assumed that the GNSS receiver uses high-quality OCXO for the current simulation scenario.

**Figure 12:**
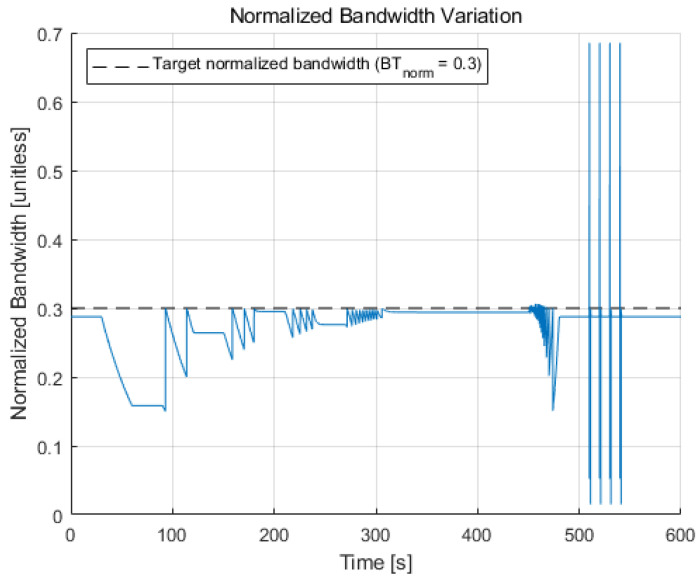
Normalized bandwidth variation during the simulation. The normalized bandwidth is maintained below the target normalized bandwidth value (i.e., 0.3) most of the time, except for the high dynamics region due to the unavoidable limitation of the integration time by the lower bound condition.

The resulting normalized bandwidth variation during the simulation is shown in Figure 12. Most of the time, the normalized bandwidth is kept below the target normalized bandwidth, which is a predefined constant value that is set to 0.3 for this paper. However, it exceeds the target value for the high dynamics region because the integration time is limited to the lower bound while the noise bandwidth is widened. The maximum value of the normalized bandwidth is approximately 0.69, which is still less than one. Therefore, despite the exceeded normalized bandwidth value, the loop filter does not lose stability.

**Figure 13:**
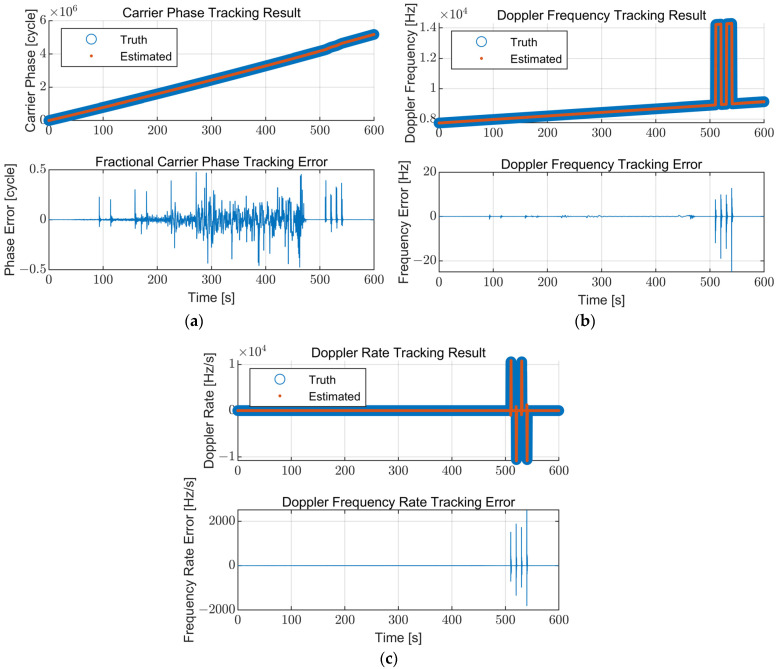
Carrier tracking results of the proposed table-based adaptive DPLL algorithm: (**a**) Carrier phase; (**b**) Doppler frequency; (**c**) Doppler rate. The proposed algorithm stably tracks the signal components for the simulation scenario.

The carrier tracking results of the proposed table-based adaptive DPLL algorithm are presented in Figure 13. The true and estimated values, including the errors between them, of the carrier phase, the Doppler frequency, and the Doppler rate are illustrated. Figure 13 shows that the proposed algorithm tracks the signal components well without the loss of lock for the whole simulation scenario. The carrier phase jitter is increased when the C/N_0_ is low. The errors in the Doppler frequency and Doppler rate increase temporally for the high dynamics region and converge to zero after the jerk dynamic stress disappears.

**Figure 14:**
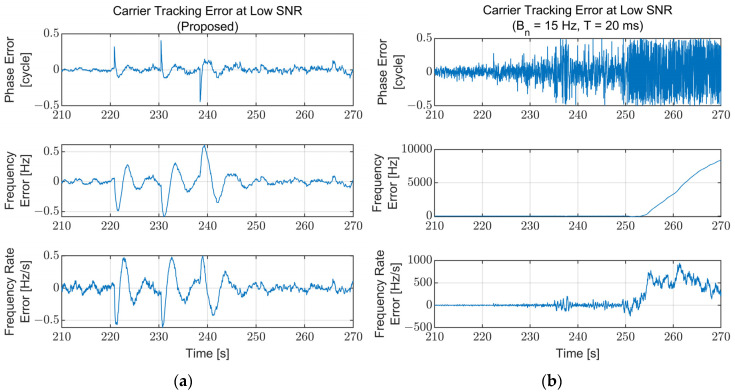
Carrier tracking error at the low SNR region (210–270 s): (**a**) Proposed algorithm; (**b**) Fixed bandwidth (B_n_ = 15 Hz, T = 20 ms). The fixed-bandwidth loop loses lock at approximately 253 s (C/N_0_ = 17 dB-Hz) while the proposed algorithm maintains its lock.

**Figure 15:**
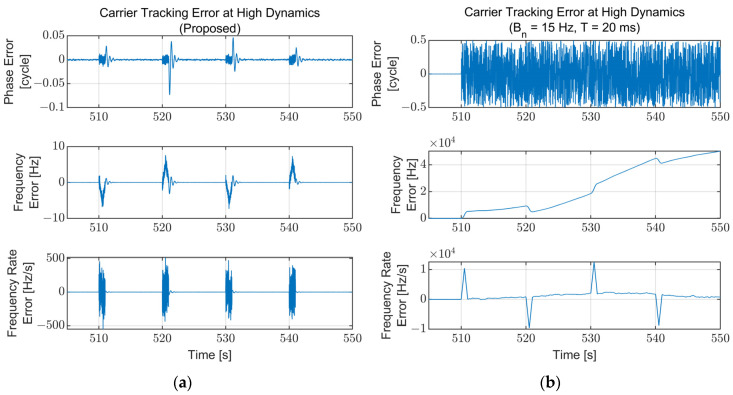
Carrier tracking error at the high dynamics region (500–550 s): (**a**) Proposed algorithm; (**b**) Fixed bandwidth (B_n_ = 15 Hz, T = 20 ms). The fixed-bandwidth loop loses lock at approximately 510 s immediately after the jerk dynamic stress occurs. The error for the proposed algorithm increases temporally while the jerk dynamic stress exists and converges to zero immediately after disappearing.

To compare the performance of the proposed algorithm with the conventional tracking loop, the carrier tracking results of a fixed bandwidth for the same simulation scenario are presented and compared with the results of the proposed algorithm. The parameters for the fixed-bandwidth loop are fixed at a noise bandwidth of 15 Hz and an integration time of 20 ms, which are typical values. Each carrier tracking error at the low SNR region (210–270 s) for the proposed algorithm and the fixed-bandwidth loop are illustrated in Figure 14. The fixed-bandwidth loop loses lock at approximately 253 s due to the low signal power (C/N_0_ = 17 dB-Hz), while the proposed algorithm maintains its lock. The carrier tracking errors for the high dynamics region (500–550 s) are presented in Figure 15. The proposed algorithm continues the tracking during the high dynamics region with the temporally increased tracking error in the existence of the jerk dynamic stress. However, the fixed-bandwidth loop loses lock immediately after the first jerk dynamic stress occurs and cannot be recovered. Figure 14 and Figure 15 show that the proposed algorithm operates stably in harsh environments where the conventional fixed-bandwidth tracking loop loses lock.

## 6. Evaluation

The proposed table-based adaptive DPLL algorithm is evaluated by comparing it with the other existing adaptive DPLL algorithms. Arranged in [3], the selected algorithms for comparison are the FAB, FL, and LBCA, which are adaptive DPLL algorithms that continually adjust the noise bandwidth of the DPLL in accordance with the SNR and the dynamic stress in an optimal manner. These algorithms are implemented in the software receiver and evaluated based on two aspects: the phase jitter performance and the computational complexity.

**Figure 16:**
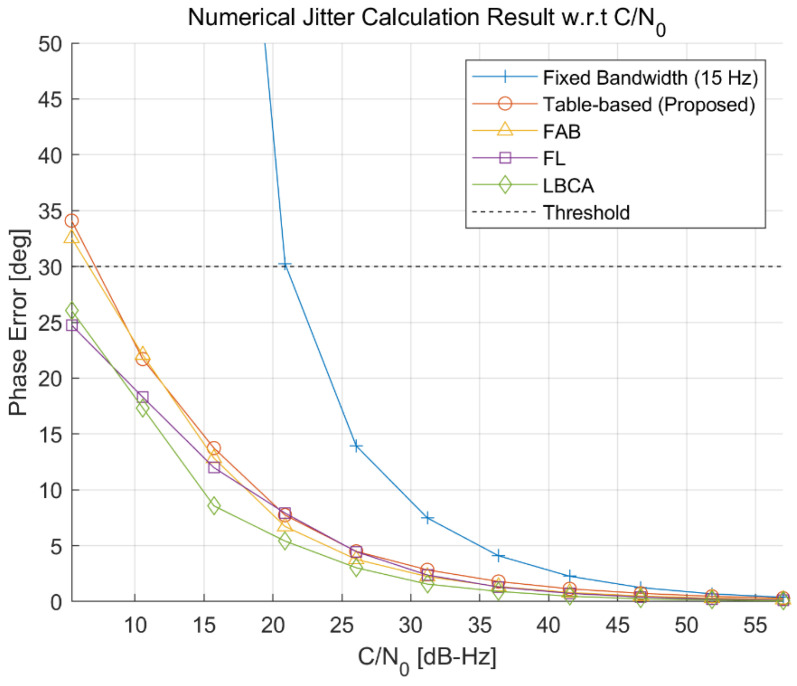
Numerical jitter calculation result with respect to the C/N_0_. The proposed algorithm has a similar performance to the fast adaptive bandwidth (FAB). Fuzzy logic (FL) and loop-bandwidth control algorithm (LBCA) have slightly better performances.

**Figure 17:**
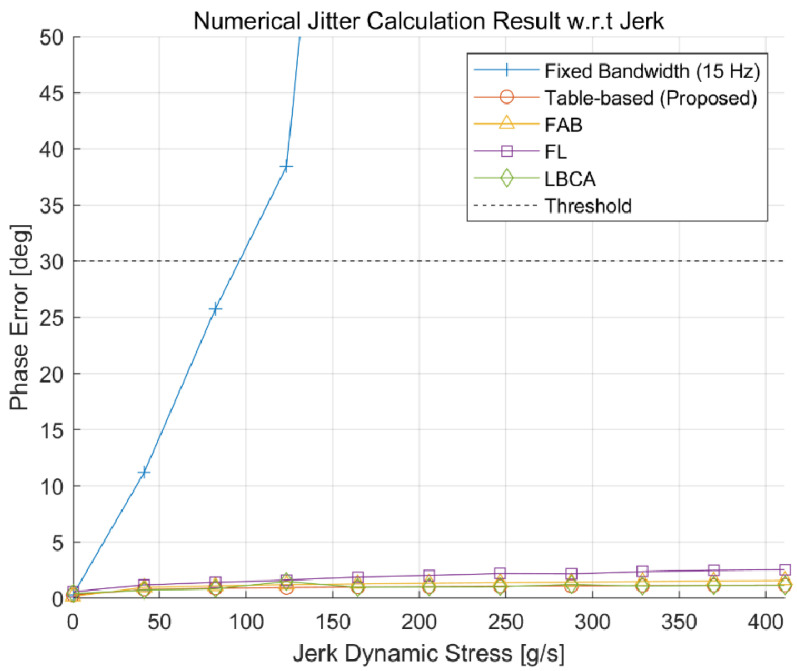
Numerical jitter calculation result with respect to the jerk dynamic stress. All adaptive DPLL algorithms have similar performances.

To compare the phase jitter performance, the standard deviation of the carrier phase tracking error for each adaptive DPLL algorithm is measured numerically for each environmental condition. Figure 16 and Figure 17 show the numerical jitter calculation results with respect to the C/N_0_ and jerk, respectively. Each point in the figures is obtained with 100 iterations of the 150 s simulation. As expected, Figure 16 shows that the fixed-bandwidth loop has the largest jitter value, and the proposed algorithm has a similar performance to the FAB. The FL and LBCA have slightly better performances than the proposed algorithm. Figure 17 shows that all the adaptive DPLL algorithms have similar performances for the jerk dynamic stress, while the fixed-bandwidth loop cannot track high dynamic signals.

**Figure 18:**
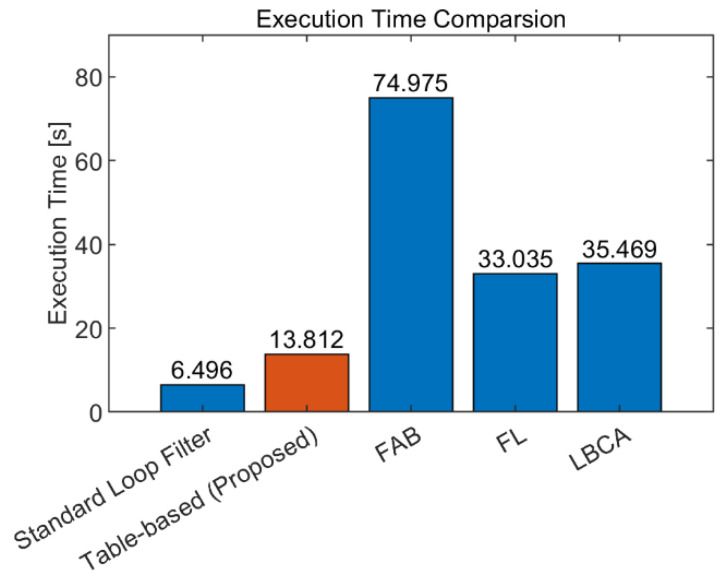
Execution time measurement results of each adaptive DPLL algorithm. The proposed algorithm has approximately 2.4–5.4 times faster execution time compared to the other adaptive DPLL algorithms.

The theoretical computational complexities of each adaptive DPLL algorithm are presented in Table 3. To concentrate on the core of the adaptive algorithms, the estimators for the SNR and the dynamic stress are excluded in this evaluation. The proposed algorithm has the smallest amount of calculation because it only contains relatively simple calculations for the determination of the table index and the calculation of the next integration time. On the other hand, the FL has the largest amount of addition (subtraction) and multiplication calculation, while the FAB and LBCA have seventh-root calculations and exponential functions that are relatively heavy operations, respectively. The execution time measurement results for each algorithm are presented in Figure 18. Each algorithm is implemented in C, and the execution time is measured using the clock() function with 10^9^ iterations for each algorithm. Each execution time result of the adaptive DPLL algorithm contains the execution time of the standard loop filter. The comparison results show that the proposed algorithm has a 2.4–5.4 times faster execution time compared to the other algorithms.

From the evaluation results presented in this section, the proposed algorithm has a similar phase jitter performance to the other existing adaptive DPLL algorithms for the various C/N_0_ and jerk dynamic stresses, while also having a faster execution time.

## 7. Conclusions

In this paper, a table-based adaptive DPLL algorithm for GNSS receivers operating in Moon exploration missions was proposed. The algorithm continually adjusts the noise bandwidth of the loop filter by measuring the current C/N_0_ and dynamic stress and extracting the noise bandwidth value from the optimal bandwidth table. The structure of the algorithm, as well as the method which calculates the proper integration time to maintain the normalized bandwidth to constant, was proposed.

The generation method and the result example of the optimal bandwidth table were presented in consideration of the thermal noise, Allan deviation oscillator phase noise, vibration-induced oscillator phase noise, and dynamic stress error. Each noise bandwidth value was selected optimally to minimize the DPLL RMSE.

The numerical simulation scenario was configured using the mission trajectory analysis results of the Moon exploration. During the simulation scenario, the C/N_0_ has a range of 5.4–57 dB-Hz, and the maximum jerk dynamic stress is 411 g/s. The proposed algorithm operated stably throughout the simulation, while a conventional fixed-bandwidth loop lost its lock. For the low SNR region, the noise bandwidth was narrowed to 0.7 Hz, and the integration time was increased to 420 ms to track the noisy signal. The noise bandwidth was widened to 213.3 Hz at the high dynamics region to track the fast variation in the signal components. Most of the time, the normalized bandwidth, which is a product of the noise bandwidth and the integration time, was maintained below the target normalized bandwidth value, so the stability of the loop filter was maintained.

The proposed algorithm was evaluated by comparing it with the existing adaptive DPLL algorithms (FAB, FL, LBCA) in terms of the phase jitter performance and the execution time. Since the noise bandwidth values in the table were generated in consideration of the optimality, the proposed algorithm has a similar phase jitter performance to the FAB and a slightly degraded performance to the FL and LBCA. However, due to the simple calculation procedure of the proposed algorithm, it has an approximately 2.4–5.4 times faster execution time compared to the other algorithms.

This paper uses the proposed algorithm for a specific scenario, the Moon exploration mission. It is suitable for the proposed algorithm because the mission has significant variations in signal power and dynamic stress during the trajectory, but they change slowly. So, adaptivity may be more important than the estimation performance of current C/N_0_ and dynamic stress. Moreover, the trajectory during the mission is fixed, so the receiver can know its approximate C/N_0_ and/or dynamic stress. Nevertheless, the proposed algorithm can be applied to any condition using a regenerated optimal bandwidth table with modified parameters. However, in the case of a situation where the signal power and dynamic stress change rapidly, sophisticated algorithms estimating C/N_0_ and dynamic stress would be needed.

## Figures and Tables

**Table 1:** Clock parameters for oven-controlled crystal oscillator [26].

Parameter	Value
$h_{0}$	2.51 × 10^−26^ [s]
$h_{-1}$	2.51 × 10^−23^ [s/s]
$h_{-2}$	2.51 × 10^−22^ [s/s^2^]

**Table 2:** Parameter values for the vibration-induced oscillator phase noise calculation [26].

Parameter	Value
$k_{g}$	2 × 10^−10^ [parts/g]
$G_{g}$	0.05 [g^2^/Hz]
$\omega_{1}$	25 × 2π [rad/s]
$\omega_{2}$	2500 × 2π [rad/s]

**Table 3:** Theoretical computational complexities of each adaptive DPLL algorithm.

Algorithm	Addition (Subtraction)	Multiplication	Division	Remarks
Table-based (proposed)	5	4	1	Floor(·)
FAB	1	16	5	Seventh-root
FL	15	20	3	-
LBCA	8	5	2	Exp(·)

## Data Availability

Not applicable.
